# Direct Imaging of Phase Objects Enables Conventional Deconvolution in Bright Field Light Microscopy

**DOI:** 10.1371/journal.pone.0089106

**Published:** 2014-02-18

**Authors:** Carmen Noemí Hernández Candia, Braulio Gutiérrez-Medina

**Affiliations:** 1 Program in Molecular Biology, Instituto Potosino de Investigación Científica y Tecnológica, San Luis Potosí, Mexico; 2 Advanced Materials Division, Instituto Potosino de Investigación Científica y Tecnológica, San Luis Potosí, Mexico; Glasgow University, United Kingdom

## Abstract

In transmitted optical microscopy, absorption structure and phase structure of the specimen determine the three-dimensional intensity distribution of the image. The elementary impulse responses of the bright field microscope therefore consist of separate absorptive and phase components, precluding general application of linear, conventional deconvolution processing methods to improve image contrast and resolution. However, conventional deconvolution can be applied in the case of pure phase (or pure absorptive) objects if the corresponding phase (or absorptive) impulse responses of the microscope are known. In this work, we present direct measurements of the phase point- and line-spread functions of a high-aperture microscope operating in transmitted bright field. Polystyrene nanoparticles and microtubules (biological polymer filaments) serve as the pure phase point and line objects, respectively, that are imaged with high contrast and low noise using standard microscopy plus digital image processing. Our experimental results agree with a proposed model for the response functions, and confirm previous theoretical predictions. Finally, we use the measured phase point-spread function to apply conventional deconvolution on the bright field images of living, unstained bacteria, resulting in improved definition of cell boundaries and sub-cellular features. These developments demonstrate practical application of standard restoration methods to improve imaging of phase objects such as cells in transmitted light microscopy.

## Introduction

Optical imaging systems present aberrations, diffraction effects at apertures and out-of-focus intensity contributions that result in images affected by blur. Consequently, the image of a point object is an extended, three-dimensional (3D) distribution of intensity (the point-spread function, PSF) [1]. Likewise, the image of a pure line object is the two-dimensional (2D) line-spread function (LSF) [2]. In terms of spatial frequency, the effects of blurring are characterized by the optical transfer function (OTF), the Fourier transform of the PSF. The point- and line-spread functions (and the corresponding transfer functions) are characteristic of linear, shift-invariant optical systems [3] and their knowledge provides valuable information to perform deconvolution image processing (or restoration), a powerful technique that removes blur [4].

In light microscopy, deconvolution processing has traditionally been linked to fluorescence–to the extent that the term ``deconvolution microscopy" almost always assumes this microscopy modality [5]–[8]. A main reason behind this association is that for the case of self-luminous objects one needs only to consider signal intensity, leading to a unique spread function and making deconvolution a linear process. This is not the case in transmitted light microscopy, where two spread functions are needed to describe image formation, as discussed below. In the case of fluorescence, the 3D image of an object 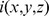 is given by the convolution of the object intensity distribution 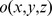 with the PSF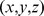:
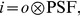
(1)where the symbol 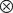 denotes the convolution operation. Therefore, the object intensity distribution can be determined (through deconvolution) if the PSF is known. Accordingly, several approaches have been developed for the evaluation of the PSF [5]–[8]. Theoretical computations of the fluorescence PSF often model light propagation along idealized imaging optics. Conversely, the PSF is experimentally measured by immobilizing on a coverslip a fluorescent bead of size below (∼1/3) the resolution limit of the microscope, followed by imaging the bead at different axial positions. The resulting stack of 2D images is the 3D PSF, which takes into account both aberration and diffraction effects present in the microscope. Despite its tremendous success and extensive use, fluorescence deconvolution microscopy requires exogenous tags and is susceptible to the effects of photostability and phototoxicity induced by the excitation light [9] during applications such as live-cell imaging.

Bright field (BF) microscopy has been proposed as an alternative to fluorescence in deconvolution image processing due to its simplicity and the possibility to observe unstained objects on a continuous basis over extended periods of time. However, its practical realization has been scarce, mainly because, unlike fluorescence, the corresponding PSF is not unique. In a classic analysis of the transmitted light microscope, Streibl showed that 3D imaging of an object with a complex index of refraction must be described by two different OTFs, each carrying separate information about absorption and phase structure [10]. Moreover, unless pure absorptive or pure phase objects are imaged, these absorption and phase responses are intertwined in BF images, precluding application of linear deconvolution procedures to remove blur. In BF, the 3D image of an object is most generally given by the sum of the convolutions of the object real (*P*) and imaginary (*A*) parts of the scattering potential with the phase (PSF_P_) and absorptive (PSF_A_) PSFs, respectively:
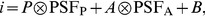
(2)where *B* is background light that did not interact with the object [10].

Previous attempts to measure the PSF_P_ in a BF microscope by imaging isolated, sub-resolution transparent beads have found it challenging due to lack of contrast [11], [12]. Limited contrast is a well-known characteristic of BF, especially during the imaging of thin, transparent (phase) objects, which become nearly invisible at exact focus. Even though phase specimens can be observed by defocusing the microscope, images are often diffuse, and features of interest show a low signal-to-noise-ratio (SNR) due to the presence of a large background arising from transmitted illumination light. To circumvent these difficulties, previous implementations of image restoration in BF [11]–[17] have mostly used stained cells or thick, opaque specimens, and rely on indirect evaluations of the spread function. In these cases, the assessed PSF is essentially the PSF_A_ since stained specimens show strong absorptive effects. Restoration experiments using staining have followed different strategies, with various degrees of success. Computational algorithms have been developed to perform blind deconvolution or PSF extraction, where a PSF is not measured but obtained concurrently with the image data [11], [12]. Alternatively, approximations to a BF PSF in deconvolution have been proposed where operation of the BF microscope is regarded similar to the fluorescence one [13], [14], a Gaussian is taken as the PSF [15], [16] or theoretical models for the corresponding OTF are employed [17].

Motivated by the need of restoration methods that: (1) are applicable to the BF imaging of phase objects such as unstained, living cells; (2) take into account the specifics of the imaging system; and (3) allow operation of conventional deconvolution routines, we address the problem of directly measuring the phase impulse responses of the transmitted light microscope and their use for deconvolution of phase objects. The main advantage introduced here is that BF imaging essentially reduces to Eq. (1) in the absence of absorption, making possible to apply linear deconvolution. In this report, we first present measurements the phase PSF (PSF_P_, hereafter referred to as the “PSF”) of a high-aperture BF microscope by direct imaging of pure phase point-objects, despite limited contrast. Our experimental approach is based on excellent SNR imaging of sub-resolution particles through computer-enhanced bright field microscopy (CEBFM), a scheme we have previously shown capable of imaging individual, unstained microtubules (MTs, slender objects only ∼25 nm in diameter) [18]. Next, we advance a simple phenomenological model of the PSF that is in excellent agreement with measurements. We also show that the corresponding OTF from measurements agrees with the result predicted by Streibl [10]. Our methodologies are further substantiated by measurement of the phase LSF (the “LSF”) of the BF microscope by using two different methods, results that confirm a well-known relationship between the PSF and the LSF in linear systems. Finally, the experimental PSF is used to perform conventional deconvolution on the BF images of living, unstained bacteria, showing significant improvement in image contrast and definition of cell boundaries.

## Results and Discussion

### Measurement of the Phase PSF

Our custom-built BF microscope has been fully described [18]. Briefly, we designed and constructed a most basic inverted microscope of high numerical aperture (NA), composed of a light-emitting diode (LED) illumination source (peak wavelength 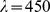 nm), collector, condenser, objective and camera lenses, and field and condenser diaphragms. The microscope is fitted with a *x*-*y*-*z* piezoelectric stage that positions the sample with ∼1–nm accuracy. Images are acquired with a 8-bit charge-coupled device (CCD) camera and transferred to a computer for the digital processing that removes background, increases contrast and minimizes noise (CEBFM, see Materials and Methods). The objective lens (100×, NA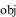 = 1.3, oil immersion) and all the microscope optics are aligned to Koehler illumination. Image contrast is maximized by reducing the condenser numerical aperture to a minimum (NA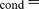 0.1 in this work, except where indicated).

**Figure 1:**
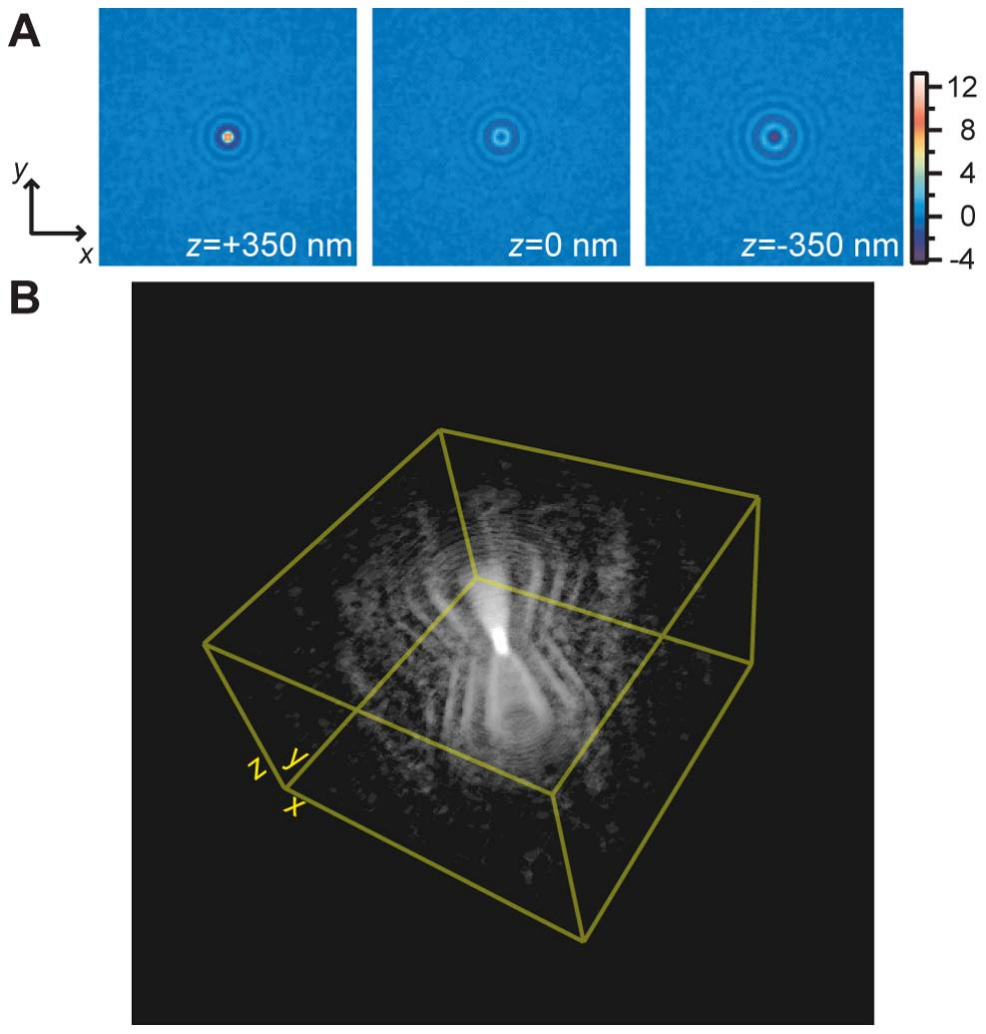
Direct measurement of the BF-PSF. (A) False-color images of a 100-nm bead at various axial positions. Field of view is 8.1 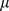
m
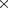
8.1 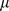
m. (B) 3D view of the measured PSF. Arbitrary transparency and threshold levels were applied for display purposes. Field of view is 8.1 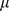
m
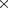
8.1 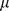
m
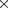
3.5 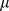
m.

To measure the PSF, we follow standard procedures used in fluorescence microscopy [19]. Polystyrene beads of 100 nm in diameter are dispersed in a salt buffer and immobilized on a coverslip, where an individual, isolated bead is imaged. BF-imaging of 200-nm beads under a moderate NA ( = 0.75) has been reported previously [20]. Here, to better approach the condition of a pure phase object, we use 100-nm beads. In the limit of particles small compared to the wavelength, the 100-nm beads are expected to scatter 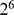 times less intensity compared to 200-nm beads [21], illustrating the difficulty of the measurements involved. To further increase the SNR, a given bead image is four-fold, rotationally-averaged by taking copies rotated by 0, 90, 180, and 270 degrees, respectively, and averaging them together. Although this last operation could discard information on possible rotational asymmetries [22], its implementation helps to better define details of the PSF (that are then compared with theory that assumes cylindrical symmetry, see below). Finally, beads are imaged at different axial positions (see Figure 1A) by moving the piezoelectric stage along the (optical) *z*-axis. Care is taken to perform measurements with adequate sampling frequencies. The best expected resolution of our microscope is 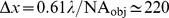 nm and 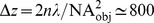 nm along the lateral and axial directions, respectively [23], where *n* ( = 1.515) is the refractive index of the medium between the specimen and the objective and condenser lenses. In our setup, each CCD pixel images an area of 68 nm×68 nm, whereas we take images along the *z*-axis spaced by 50 nm, thus satisfying the Nyquist criterion of sampling at intervals of at least half the resolution distance [24].

**Figure 2:**
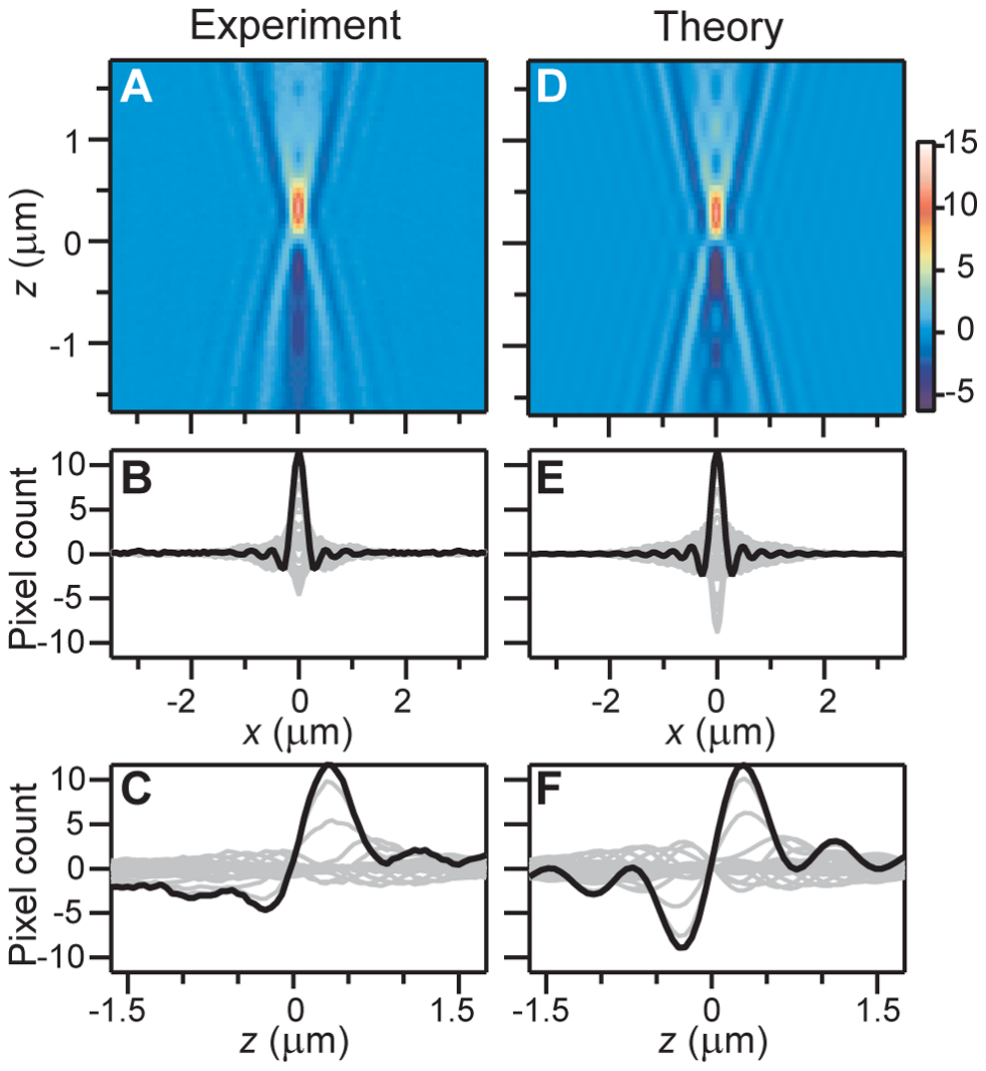
Main characteristics of the PSF and comparison with theory. (A) False-color, vertical slice (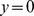) of the experimental PSF. (B) Superimposed lateral cross-section profiles (light gray). (C) Axial cross-section profiles. (D) Vertical slice (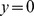) of the theoretical PSF. (E) Lateral cross-section profiles. (F) Axial cross-section profiles. The intensity of the theoretical model was multiplied by an arbitrary factor for comparison with experiment. Profiles highlighted in black correspond to the maximum positive intensity peak of the PSF, used to determine the PSF main spot size.

The emerging 3D PSF (see Figure 1B) and a representative central *x*-*z* slice (Figure 2A) feature a high peak SNR (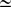
100, see Materials and Methods), allowing for several interference-diffraction fringes to be clearly distinguished. Although the hourglass-like aspect is similar to its fluorescence counterpart, the BF-PSF has distinctive characteristics, showing negative and positive intensity count values, together with a main central lobe that changes from negative to positive amplitude as the 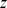-position is changed (i.e. as the microscope is defocused). This last effect is, as expected, due to the 100-nm polystyrene bead acting as an effective phase object being small (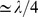) and mostly transparent at the wavelength range involved. For reference purposes, we define here a 3D coordinate system where *x* = 0, *y* = 0 locates the center of the bead on the image plane, and *z* = 0 locates the axial position where the phase object is the least visible. Using these definitions, pixel count vs. *x*, *z* profiles from the central *x*-*z* slice of the PSF further show good fringe visibility (see Figure 2B) and an (axially) asymmetrical central lobe with a larger positive amplitude (see Figure 2C). The widths of the main spot are 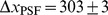 nm and 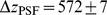 nm, along the *x*- and *z*-axis, respectively (see Figure S1).

### Phenomenological Model of the Phase PSF

A simple phenomenological model can help understand the main specifics of the measured BF-PSF. According to Abbe's theory of the microscope, interaction of illumination light with an object results in diffracted (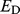) and non-diffracted (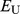) field components, which give rise to the image intensity distribution (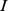) upon interference at the image plane [24], [25]: 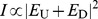. The term 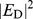 is negligible here, as the scattered amplitude from the phase object is small compared to the illumination wave, whereas the term 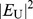 is absent in our background-free images. Therefore, we detect only the interference term 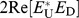. In addition, the non-diffracted component has constant amplitude in BF under Koehler illumination (as it gives rise to an even background) while the diffracted component is retarded in phase by 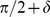 with respect to 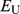, where 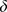 is the phase change introduced by the object due to differences in optical path length with the surrounding medium [26]. For the case of our sub-resolution beads, we approximate 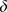 by a constant, effective phase shift. Furthermore, for NA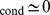 (approximately the value used in our experiments), 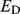 at the image plane has an amplitude form corresponding to the field distribution of a point source, 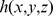, which is already known from fluorescence microscopy for the case of an ideal microscope with rotationally symmetric pupils and sources [5]:
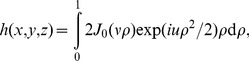
(3)where 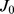 is the zeroth-order Bessel function of the first kind, 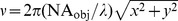, and 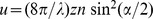. Here, NA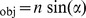.

Taking together the considerations above, the expected intensity distribution of the BF-PSF on the image plane is



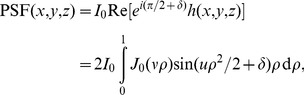
(4)where 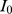 is the peak intensity. The mean phase retardation induced by a bead of radius 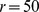 nm is 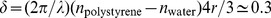. To compare with the experimental data, we use the experimental value NA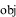 = 1.3 for scaling along the 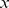-axis (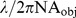), and 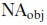 = 1.24 for scaling along the 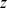-axis (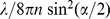). A graph of Eq. (4) (setting 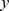 = 0) is displayed in Figure 2D, together with its corresponding profiles (see Figures 2E, 2F). The widths of the main spot of the theoretical PSF are 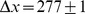 nm and 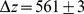 nm (see Figure S1). The theoretical PSF shows excellent agreement with the experimental result. Differences in the NA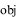 value used for scaling along 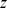 and in the smaller negative count values of the measured PSF with respect to the model (see Figures 2C, 2F) are attributed mainly to spherical aberration due to the bead being located at the glass-water boundary.

**Figure 3:**
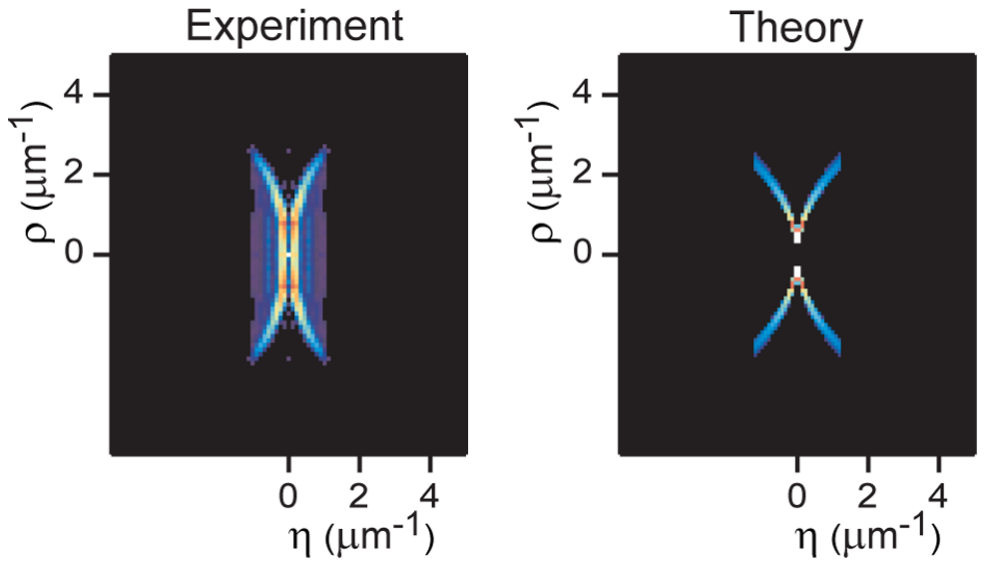
Experimental OTF and comparison with theory. The modulus of the OTF is displayed (left) together with the result predicted by Eq. (5) (right), showing good agreement.

These results can be compared with the expected phase OTF for the transmitted light microscope. According to Streibl [10], the phase OTF (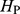) for the case of a circular illumination source and a circular imaging pupil is:
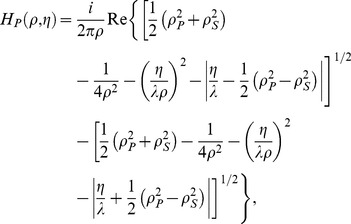
(5)where 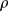 and 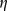 denote the radial and axial spatial frequencies, respectively, and 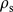 and 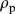 are the magnitude of the greatest lateral component of beam wavevectors illuminating the object and the maximum spatial frequency allowed by the imaging pupil, respectively. To compare with this prediction, we obtain the experimental OTF by computing the 2D FFT of the PSF shown in Figure 2A, whereas the theoretical OTF is determined by Eq. (5), setting 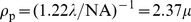
m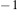 and 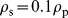 (the regime of almost coherent illumination, NA
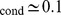
NA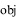, used here). Figure 3 shows good agreement between the experimental and theoretical OTFs, confirming earlier predictions. We conclude this section by noting that our measured PSF can be regarded as obtained by a single-beam BF interferometer, where the forward scattered field is detected in the far field upon interference with the non-scattered (reference) wave [27].

### Measurement of the Phase LSF

**Figure 4:**
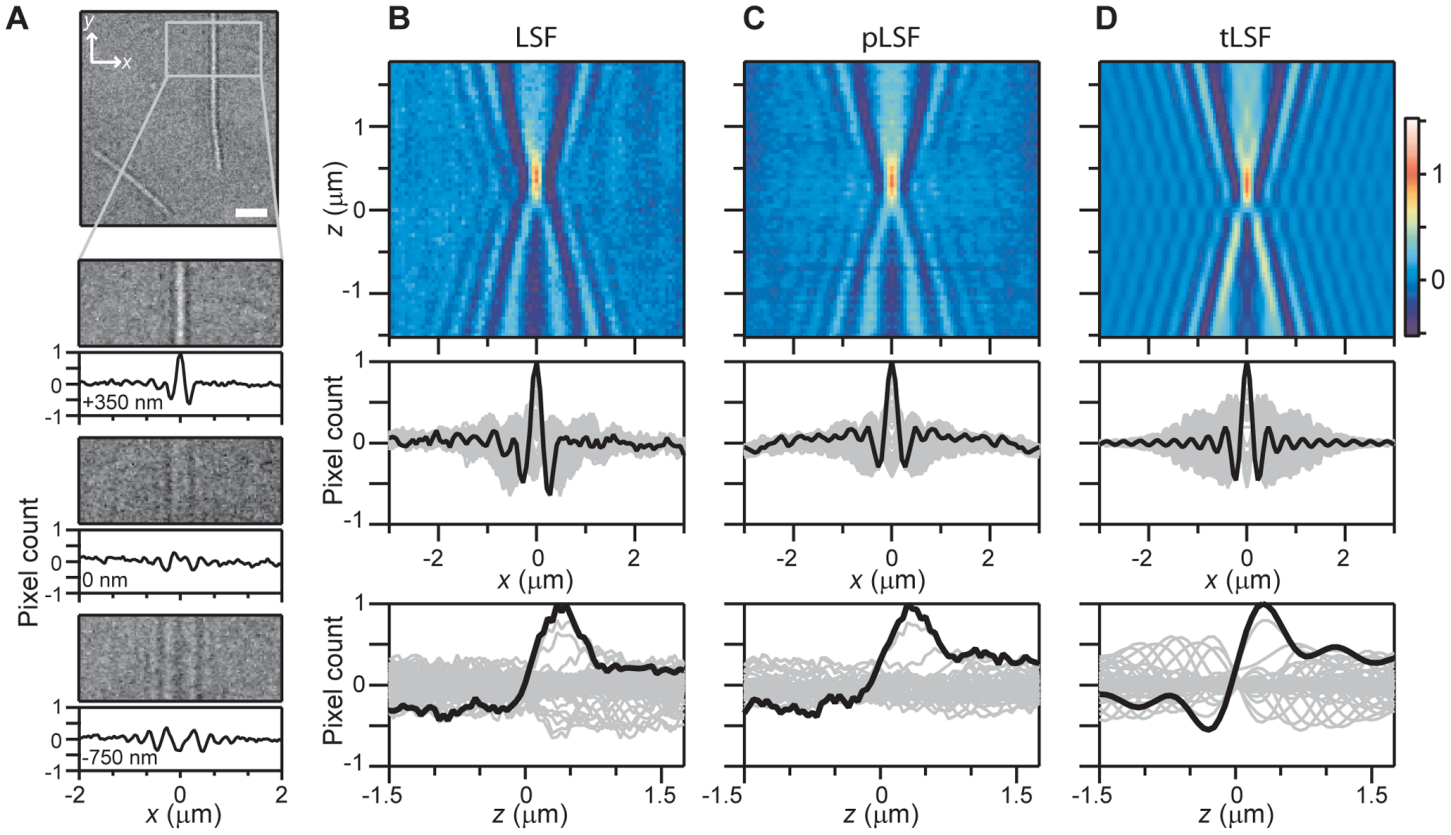
Direct and indirect measurements of the BF-LSF. (A) A straight segment of an individual MT is chosen, shown at various defocusing positions. The corresponding pixel count profiles were obtained for each image by averaging all the pixel count values along a given pixel column. Scale bar: 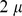
m. (B) The directly-measured LSF obtained from MT-profiles (false-color), together with intensity profiles. (C) The indirectly measured pLSF obtained from the experimental PSF (false-color), together with intensity profiles. (D) The tLSF derived from the theoretical PSF, together with intensity profiles. Profiles highlighted in black were used to determine main spot sizes. The intensities of the pLSF and tLSF were multiplied by arbitrary factors for comparison with the LSF.

To further validate our approach and explore the impulse responses of the BF microscope, we decided to measure the phase LSF by imaging individual, unstained MTs. MTs are the biological polymer filaments (∼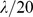 in diameter, several micrometers in length) involved in cellular structure and organization [28]. MTs were immobilized on coverslips as described [18], and imaged at various axial positions, as with beads (see Figure 4A). Straight, middle MT sections were selected for analysis to avoid end effects. To improve SNR, pixel averages along the MT direction were evaluated. The collection of these intensity profiles along the 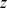-axis is the LSF, directly measured using our optical microscope (see Figure 4B). Similarly to the measured PSF, the LSF shows a set of clearly defined interference-diffraction fringes (peak SNR 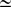 19), whose intensities are below the single-count value of the 8-bit CCD camera–a remarkable demonstration of the capabilities of CEBFM. Contrary to the PSF case, however, profiles of the LSF corresponding to different defocusing distances (see Figure 4B) reveal broad distributions conformed by secondary fringes that are comparable in intensity to the primary, central spot, and whose decay away from the center along both lateral and axial directions is slow. Additionally, we find that the main spot of the LSF has smaller lateral width (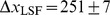 nm) but larger axial width (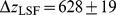 nm) compared to the measured PSF (see Figure S2).

The previous measurements provided us with an opportunity to experimentally verify a known relationship between the LSF and the PSF
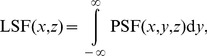
(6)valid in linear systems, for a line excitation along the *y*-axis [1]. To this end, a LSF (which we call pLSF) was obtained simply by adding all pixel counts of the experimentally measured PSF (see Figure 1B) along the *y*-axis. The pLSF thus obtained (see Figure 4C) compares well with the measured LSF (using MTs). The widths of the main spot of the pLSF are 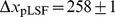 nm and 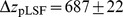 nm (see Figure S2), consistent with the values from the directly measured LSF. Furthermore, a theoretical LSF (tLSF, see Figure 4D) generated by adding intensity counts along the *y*-axis of the PSF from the phenomenological model yields 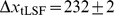 nm and 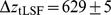 nm (see Figure S2). These results confirm that the lateral (axial) width of the LSF is intrinsically smaller (larger) by about 15% compared to that of the PSF.

### Conventional Deconvolution Processing of BF Images

The high-SNR phase PSF measured, substantiated by our modeling and the evaluations of the phase LSF, constitutes an excellent starting point to perform standard deconvolution processing in BF. As noted before, conventional, linear deconvolution can indeed be applied to pure phase objects provided the phase PSF is used [10]. Although a number of mechanisms of interaction between illumination light with the specimen (such as refraction and multiple scattering besides absorption and phase variations) can influence BF microscopy images to various extents, living, unstained cells can be regarded as pure phase objects to a good approximation. *Escherichia coli* cells immersed in growth medium were deposited on coverslips and allowed to sediment, after which samples were taken to the BF microscope for visualization. Cell image *z*-stacks were acquired as with beads. To reduce multiple fringe superpositions, we imaged cells at NA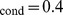, and a corresponding PSF at this numerical aperture of the condenser was acquired and used for deconvolution. We carried out 3D restoration of image stacks using the ImageJ plugin “Iterative Deconvolve 3D”, which computes non-negative amplitude, iterative deconvolution [29] (see Materials and Methods). As a first, trivial check, deconvolution of the reference PSF with itself results in an expected single spot centered around 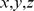 = 0 (see Figure S4).

**Figure 5:**
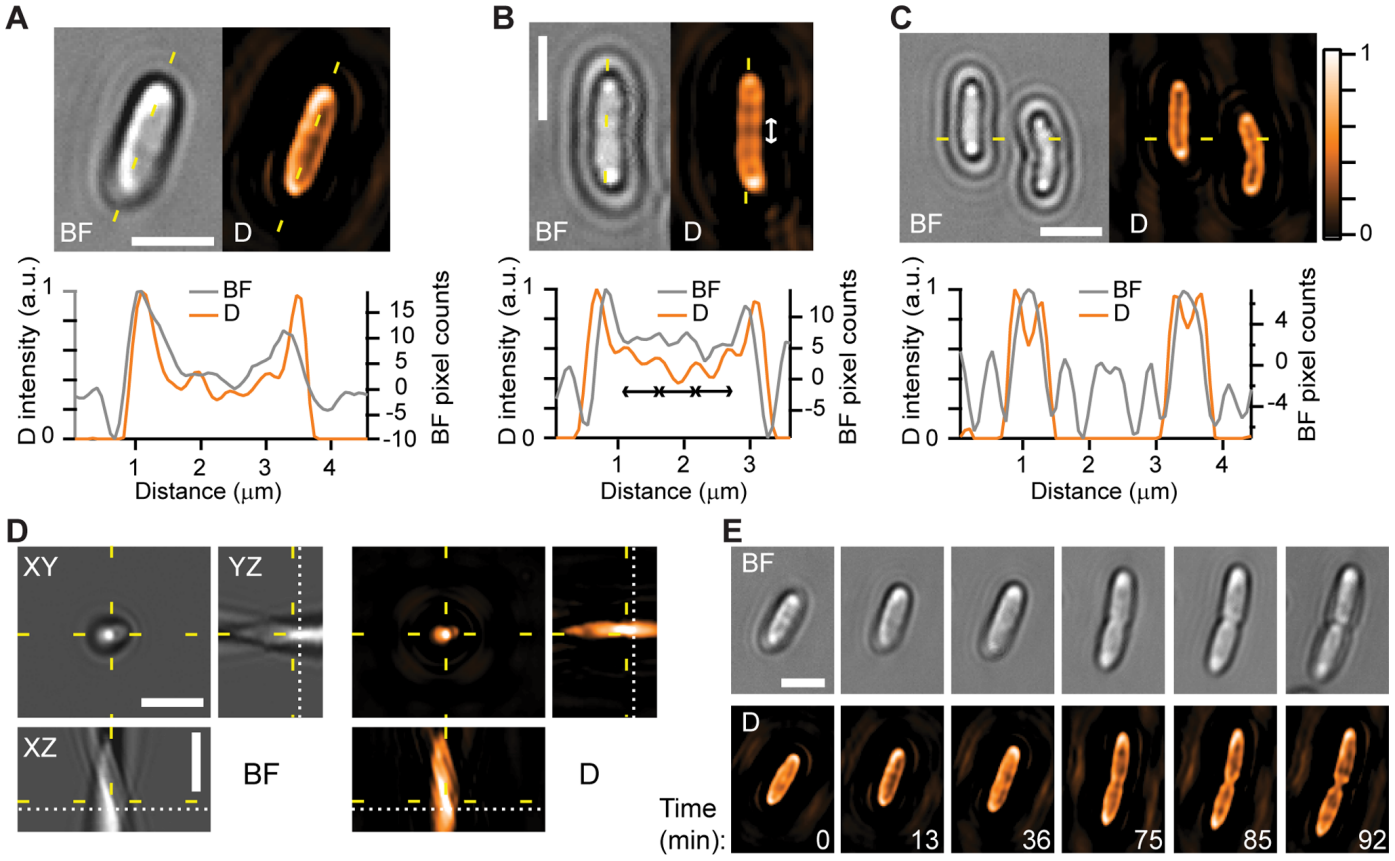
Demonstration of deconvolution in the BF microscopy images of unstained, living *E. coli* cells. (A–C) BF images of bacteria before (“BF”) and after (“D”) deconvolution, together with their respective intensity profiles along the yellow dashed lines. Length of double-arrow lines in (B): 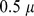
m. (D) Image of a standing bacterium along three orthogonal slices (marked by the yellow dashed lines). In BF, the cell extends well beyond the position of the supporting coverslip (white dotted line), whereas the same views after deconvolution display the cell with improved definition of boundaries. (E) Time-lapse frames of a bacterium undergoing cell division under continuous illumination. No threshold or transparency levels were applied to deconvolved images. Scale bars: 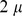
m.

We next performed deconvolution of cell frames. One notoriously adverse aspect of BF images is that they present variations between negative and positive intensity values due to the strong influence of defocusing and out-of-focus scattered light, making difficult to identify object locations and boundaries. In contrast, the deconvolved frames show significant improvement in clarity (see Figures 5A–C), where the ambiguity of regions changing in intensity from positive to negative values is removed. In particular, cell wall boundaries become well defined, displaying a striking resemblance to fluorescence microscopy images of labelled cells [30]. Likewise, intensity variations along the cell body that are only hinted in the original BF frames gain contrast after deconvolution. Some of these variations (see Figure 5B) show a degree of spatial periodicity (∼0.5 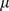
m) and may correspond to the same structures observed recently in unstained bacteria using dSLIT, a microscopy technique capable of quantitative phase imaging [31]. Here, sensitivity to phase variations caused by the object is expected, as evidenced by Eq. (4). Image improvement is also observed in *z*-stacks, as demonstrated by performing deconvolution on the images of a bacterium whose orientation is standing above the coverslip (see Figure 5D). In the original BF images, the location and boundaries of the bacterium are difficult to distinguish, and the bacterium body appears to extend well into the supporting coverslip. These problems are much reduced after deconvolution, where cell orientation and boundaries are better defined.

Finally, one attractive feature of BF deconvolution is the potential to observe unstained specimens over extended periods of time. We show this aspect by following changes in *E. coli* shape as cell division proceeds under continuous illumination (see Figure 5E). Using deconvolution, cell walls, internal structure and the development of the septum at mid-cell all become clearly defined. Therefore, the methodologies presented here could prove useful in quantifying cell shape and internal dynamics over many division cycles on the same cells on a continuous basis.

### Conclusions

The results presented here introduce practical methodologies in BF microscopy to directly measure the corresponding phase spread functions, from where conventional deconvolution processing is demonstrated. Our procedures are applicable to the imaging of thin, transparent speciments such as living, unstained cells. Future developments include using a camera with increased bit depth (to enhance sensitivity and response times) together with evaluations to recover quantitative information on optical path variations from BF images, similarly to recently developed quantitative phase microscopy techniques [32], [33].

## Materials and Methods

### Bead Samples

Microscope slides and coverslips were cleaned prior to use for 5 min in a plasma cleaner (Harrick Plasma) at 1 Torr (ambient air). Flow channels were made using double-sided tape as described [18]. Polystyrene beads of 100 nm in diameter (Invitrogen, F8803) were diluted 1∶100 from the stock in miliQ water and sonicated during 10 min, followed by a second 1∶100 dilution in miliQ water and sonication over additional 10 min. After a final 1∶100 dilution in HEPES buffer (50 mM HEPES, 10 mM MgCl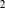, pH 7.5) beads were introduced into a flow channel and allowed to bind to the coverslip.

### MT Samples

Tubulin (TL238-C, Cytoskeleton) was polymerized to produce MTs as described [18]. To immobilize MTs on coverslips, flow channels were prepared using poly-L-lysine-coated coverslips. A rack of plasma-cleaned coverslips was submerged for 15 min in a solution of 600 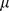
L of poly-L-lysine diluted in 300 mL of ethanol, oven dried at 40°C, and stored. Stabilized MTs were diluted in PEMTAX buffer (0.02 mM Taxol, 80 mM PIPES, 1 mM EDTA, 4 mM MgCl_2_, pH 6.9), introduced into the flow channel and incubated over 10 min. Unbound MTs were removed by washing channels with 40 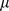
L of PEMTAX buffer.

### Bacteria Samples

*E. coli* TOP10 cells were grown overnight in Luria Broth medium. A sample of a 1∶100 dilution in fresh medium was introduced into flow channels. Coverslips were used uncoated or coated with poly-L-lysine. Experiments were performed at room temperature, (22±2)°C.

### Optical Microscopy and CEBFM

We perform background subtraction and frame averaging on all our images. To eliminate unwanted, uneven background arising from specks of dust or reflections in lenses, a total of 250 frames are captured and averaged to produce a single background frame that is subsequently subtracted from all incoming frames. Background subtraction is further optimized by displacing the microscope stage in 3D (along non-closed paths covering distances of a few micrometers) while background frames are taken. This last action is performed with the piezoelectric stage on which the sample is mounted, and has the effect of averaging out intensity contributions in the final background image due to small debris found on the coverslip surface. We reduce electronics noise by arithmetical averaging of 50 background-free frames, producing a single low-noise, high-contrast image of a given subject at a specified *z*-position. A typical *z*-stack of 70 frames is acquired in ∼2.5 min, and stored as a set of text files for further processing/analysis. During processing, the original 8-bit images are converted to 16-bit and carried out in that form throughout. Image acquisition and digital processing was performed using LabView 8.5 (add-on package Vision, National Instruments).

### PSF, LSF Image and Data Analysis

To obtain the phase PSF and phase LSF from bead and MT images, respectively, we first select regions-of-interest. In the case of the PSF, a given bead image is four-fold, rotationally-averaged, after which a mean filter of 0.5 pixels in radius is applied. These operations are performed using ImageJ [34]. Next, profile curves are generated from central slices of the PSF or from MT images in the LSF case. The pLSF was obtained by adding pixel counts along the *y*-axis of the MT-measured LSF and scaling the intensity appropriately. Similarly, the theory model tLSF was generated by adding intensity counts along the *y*-axis of the theoretical PSF (whose central slice is shown in Figure 2D), and the intensity was scaled appropriately. We estimate the peak SNR in our measurements as the ratio of the maximum, positive pixel count value of the central spot in the PSF (LSF) divided by the standard deviation value of residual background noise in an arbitrary, nearby 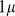
m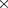 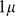
m region where no bead (MT) is present. FFT analysis of the measured phase PSF was performed using ImageJ. Data analysis was performed using Igor Pro 5.0 (Wavemetrics).

### Measurement of the Widths of the PSF and LSF

For both the phase PSF and phase LSF, we consider the profiles corresponding to the maximum positive pixel count value (highlighted in black in Figure 2 and Figure 4). Next, we perform fits of the central section of the profiles to the following functions:
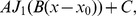
for the *x*-profiles, and



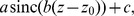
for the *z*-profiles, where 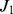 is a first-order Bessel function of the first kind, and (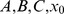) and (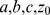) are fitting parameters. As a measure of width, for the case of the 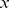-profile we take the distance from the main peak to the first adjacent minimum (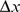), whereas for the 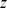-profile we take the distance between the maximum and minimum peaks (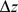). Therefore, 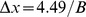, and 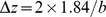 (see Figures S1, S2).

### BF Deconvolution

A phase PSF with enhanced SNR for deconvolution was obtained by performing 72-fold, rotational-averaging, where the image of a single bead was rotated in increments of 5 deg and all the rotated images were added. This operation was followed by application of a mean filter of 0.5 pixels in radius. These procedures were followed for each image of the 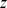-stack. Finally, all the frames in the stack were multiplied by an overall factor such that the maximum, positive intensity count value of the entire PSF was set to ∼250, as we found this was a good magnitude to perform deconvolution (see Figure S3). A substack of 41 frames in *z*, centered around *z* = 0, was used as the reference PSF during deconvolution processing throughout (see Figure S3). *z*-stacks of *E. coli* images were acquired using CEBFM (subtracting background and performing 50-frame averages to produce single frames corresponding to given *z*-positions). For each *E. coli* sample of interest, a substack consisting of 41 frames was selected for analysis, except with the sample shown in Figure 5D, where a 71-frame substack was deconvolved. Deconvolution was performed using the ImageJ plugin `Iterative Deconvolve 3D' [29], [35]. This algorithm applies a Wiener filter [36] as a preconditioning step, followed by an iterative least squares solver that sets negative values in the deconvolved image equal to zero at the end of each iteration. Therefore, this routine produces final deconvolved frames with non-negative signal amplitude. All images were deconvolved using as kernel the 41-frame, reference PSF (shown in Figure S3), together with the following parameters: Wiener filter gamma regularization parameter, 0.001; low pass filter, 1 pixel in *z*, 0 pixel in *x*; number of iterations, 30. Except for a multiplicative factor to set maximum intensity levels equal to 1, no threshold or transparency adjustments were applied to images after deconvolution. The axial position of the coverslip (see Figure 5D) was found by observing small debris on the coverslip upon defocusing of the microscope, and set as the point where debris became the least visible after being mainly bright but before being mainly dark.

## Supporting Information

Figure S1
**Finding the widths of the PSF.** Profiles corresponding to the maximum positive pixel count value (black) are fitted on the central interval (red). (a) Fits to the directly measured PSF yield 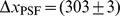 nm and 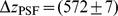 nm. (b) Fits to the theoretical PSF yield 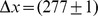 nm and 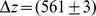 nm. Errors from the fit.(TIF)

Figure S2
**Finding the widths of the LSF.** Profiles corresponding to the maximum positive pixel count value (black) are fitted on the central interval (red). (a) Fits to the directly measured LSF (using MTs) yield 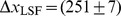 nm and 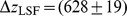 nm. (b) Fits to the indirectly measured pLSF (using the measured PSF) yield 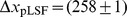 nm and 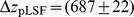 nm. (c) Fits to the tLSF derived from the theoretical PSF yield 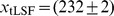 nm and 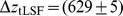 nm. Errors from the fit.(TIF)

Figure S3
**Deconvolution processing of the PSF with itself.** The central *x*-*z* slice of the measured PSF using NA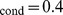, together with its corresponding intensity profiles (left column). The corresponding section of the PSF marked by the rectangle (yellow, dashed line) was taken as the reference PSF for deconvolution of bacteria images. Using the reference PSF to deconvolve the whole PSF image, results in the deconvolved PSF and corresponding profiles (right column). The deconvolved image of the 100-nm bead is centered around the point 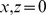, as expected. Profiles highlighted in black correspond to the maximum intensity point of the PSF or the deconvolved PSF. The widths of the highlighted profiles for the deconvolved PSF are: FWHM  = 260 nm (*x*) and FWHM  = 420 nm (*z*).(TIF)
