# Supplementary figures and images for: Direct Imaging of Phase Objects Enables Conventional Deconvolution in Bright Field Light Microscopy
Source: PLoS One. 2014 Feb 18;9(2):e89106. doi: 10.1371/journal.pone.0089106 (PMC3928359; doi:10.1371/journal.pone.0089106)

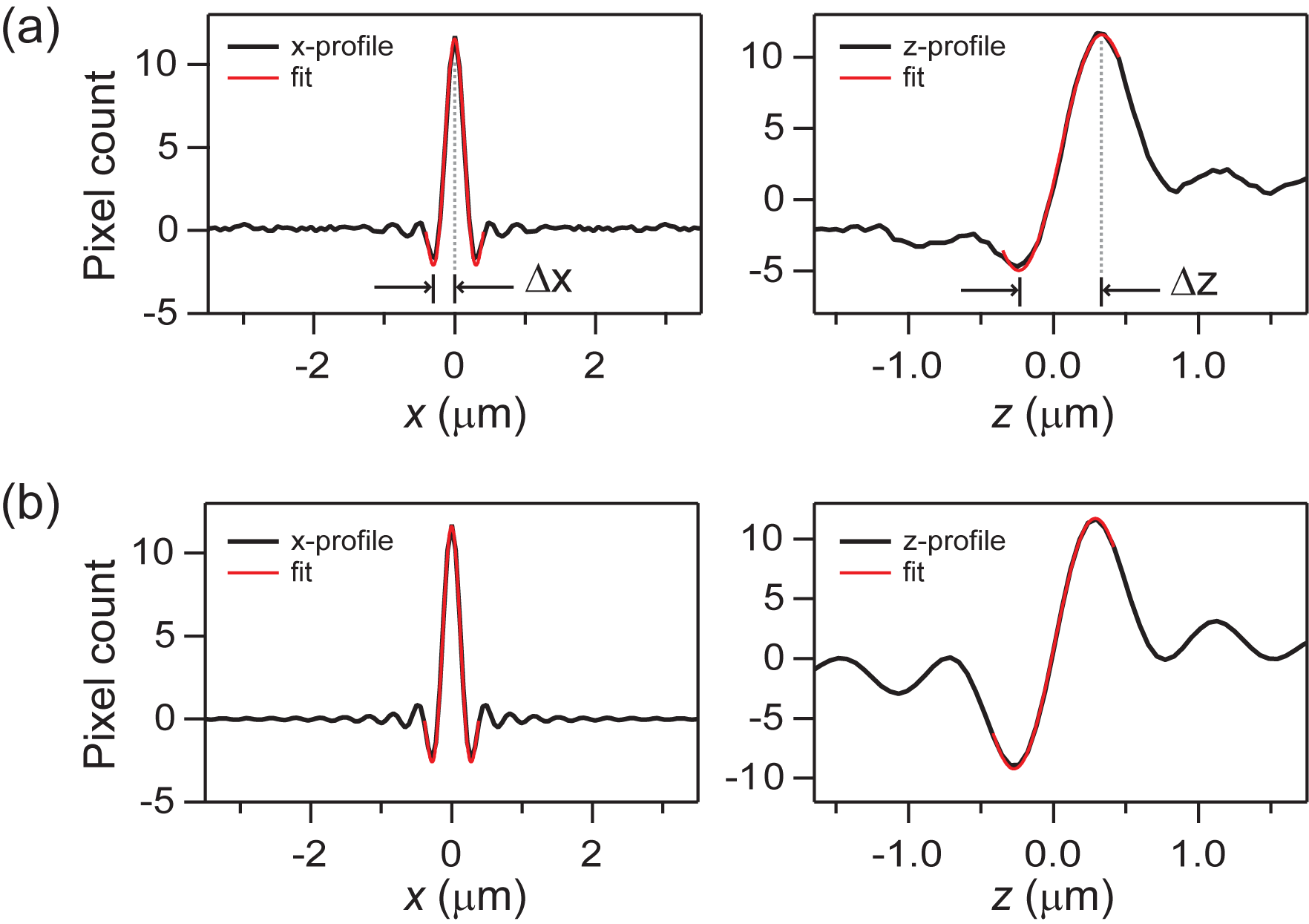

Supplement: Figure S1 — Finding the widths of the PSF. Profiles corresponding to the maximum positive pixel count value (black) are fitted on the central interval (red). (a) Fits to the directly measured PSF yield nm and nm. (b) Fits to the theoretical PSF yield nm and nm. Errors from the fit. (TIF) [file pone.0089106.s001.tif]

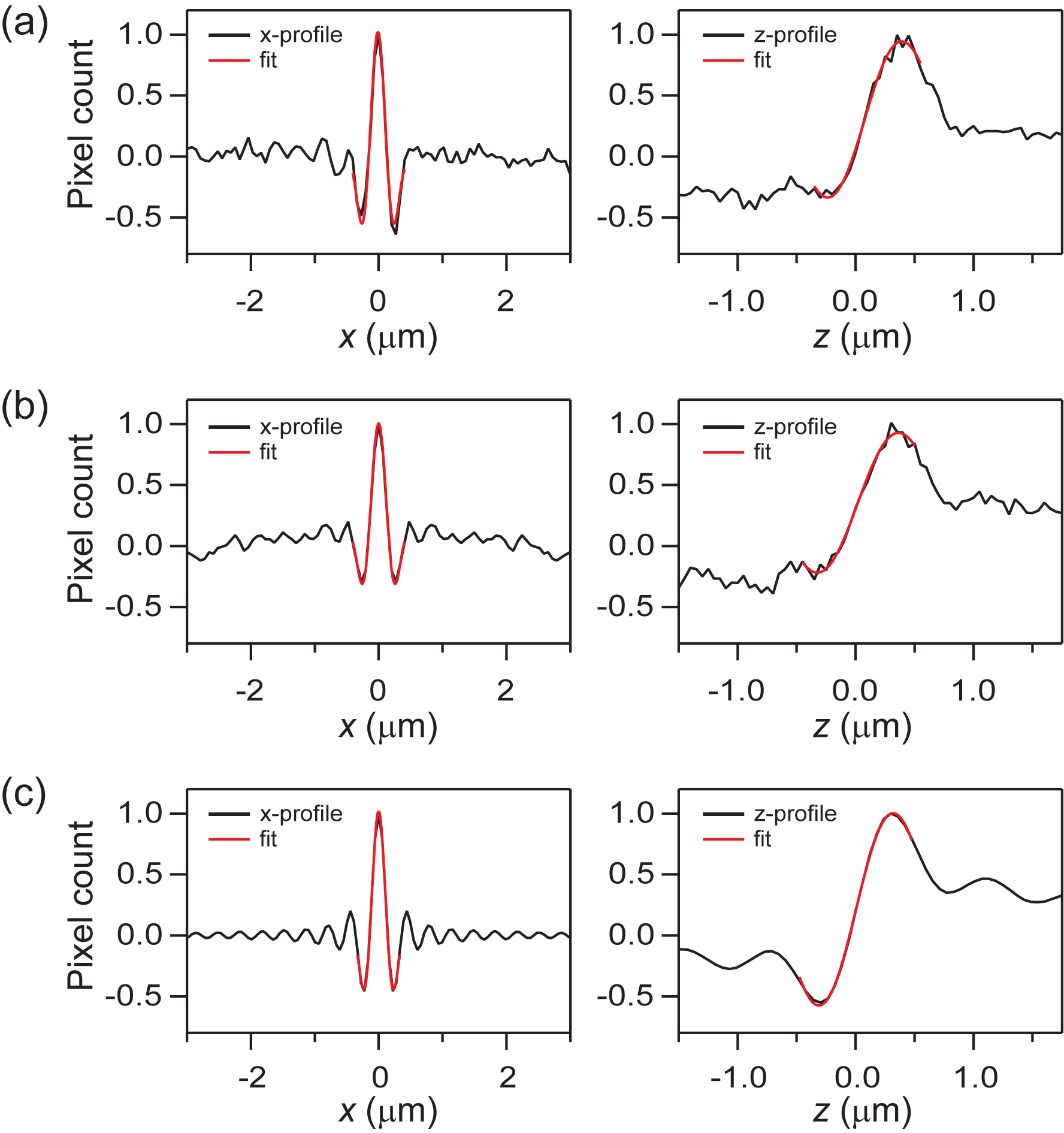

Supplement: Figure S2 — Finding the widths of the LSF. Profiles corresponding to the maximum positive pixel count value (black) are fitted on the central interval (red). (a) Fits to the directly measured LSF (using MTs) yield nm and nm. (b) Fits to the indirectly measured pLSF (using the measured PSF) yield nm and nm. (c) Fits to the tLSF derived from the theoretical PSF yield nm and nm. Errors from the fit. (TIF) [file pone.0089106.s002.tif]

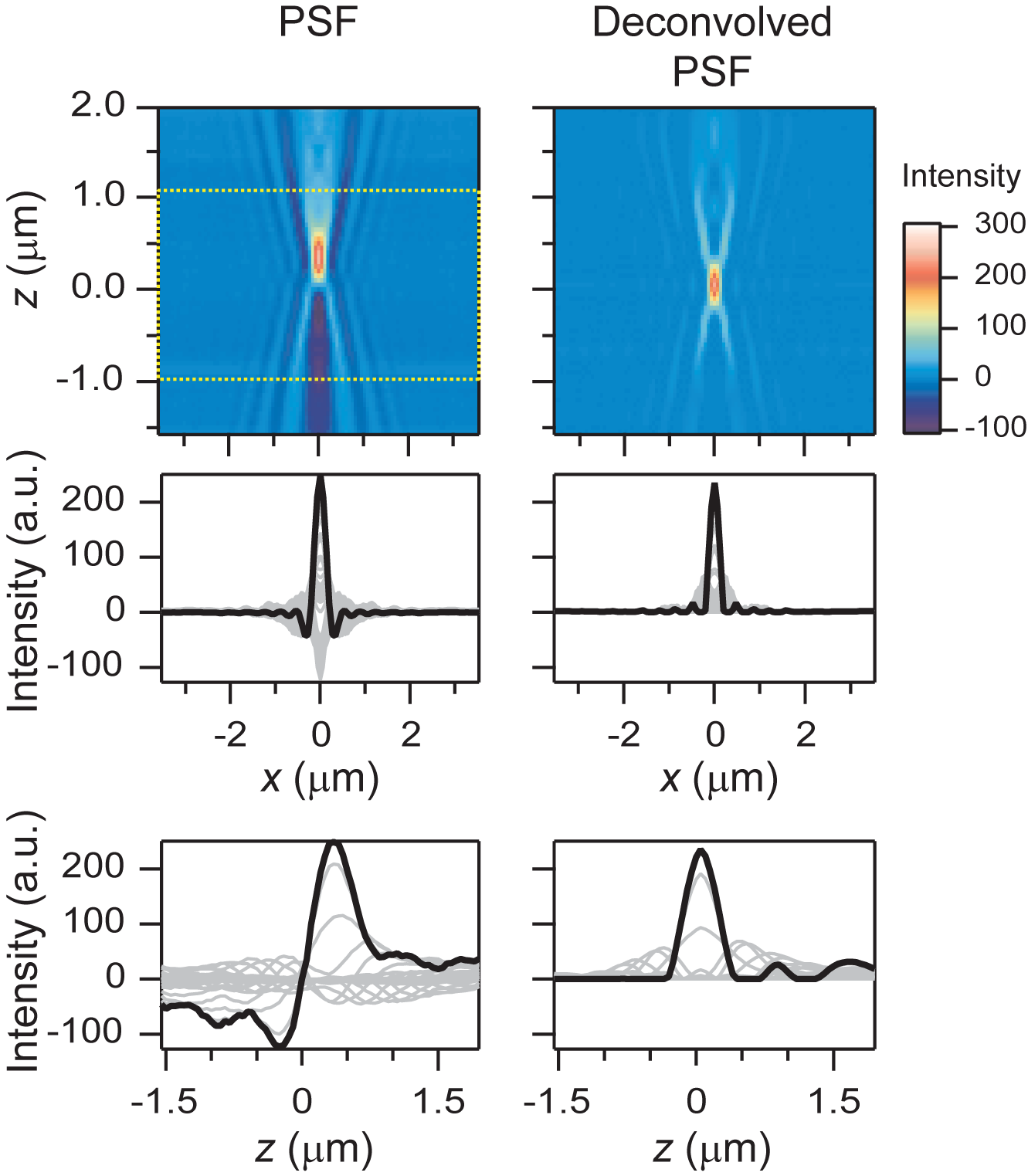

Supplement: Figure S3 — Deconvolution processing of the PSF with itself. The central x-z slice of the measured PSF using NA, together with its corresponding intensity profiles (left column). The corresponding section of the PSF marked by the rectangle (yellow, dashed line) was taken as the reference PSF for deconvolution of bacteria images. Using the reference PSF to deconvolve the whole PSF image, results in the deconvolved PSF and corresponding profiles (right column). The deconvolved image of the 100-nm bead is centered around the point , as expected. Profiles highlighted in black correspond to the maximum intensity point of the PSF or the deconvolved PSF. The widths of the highlighted profiles for the deconvolved PSF are: FWHM = 260 nm (x) and FWHM = 420 nm (z). (TIF) [file pone.0089106.s003.tif]
